# Femoral Neck Thickness Index as an Indicator of Proximal Femur Bone Modeling

**DOI:** 10.3390/vetsci10060371

**Published:** 2023-05-24

**Authors:** Pedro Franco-Gonçalo, Ana Inês Pereira, Cátia Loureiro, Sofia Alves-Pimenta, Vítor Filipe, Lio Gonçalves, Bruno Colaço, Pedro Leite, Fintan McEvoy, Mário Ginja

**Affiliations:** 1 Department of Veterinary Science, University of Trás-os-Montes and Alto Douro, 5000-801 Vila Real, Portugal; pedrofranco@utad.pt (P.F.-G.); ines.pereira444@gmail.com (A.I.P.); 2 Animal and Veterinary Research Centre (CECAV), University of Trás-os-Montes and Alto Douro, 5000-801 Vila Real, Portugal; salves@utad.pt (S.A.-P.); bcolaco@utad.pt (B.C.); 3 Associate Laboratory for Animal and Veterinary Sciences (AL4AnimalS), University of Trás-os-Montes and Alto Douro, 5000-801 Vila Real, Portugal; 4 Department of Animal Science, University of Trás-os-Montes and Alto Douro, 5000-801 Vila Real, Portugal; 5 Department of Engineering, University of Trás-os-Montes and Alto Douro, 5000-801 Vila Real, Portugal; catialoureiro1999@gmail.com (C.L.); vfilipe@utad.pt (V.F.); lgoncalv@utad.pt (L.G.); 6 Institute for Systems and Computer Engineering (INESC-TEC), Technology and Science, 4200-465 Porto, Portugal; 7 Neadvance Machine Vision SA, 4705-002 Braga, Portugal; pleite@neadvance.com; 8 Department of Veterinary Clinical Sciences, Faculty of Health and Medical Sciences, University of Copenhagen, 1870 Copenhagen, Denmark; fme@sund.ku.dk

**Keywords:** dog, canine hip dysplasia, femoral neck thickness index, bone modeling, osteoarthritis

## Abstract

**Simple Summary:**

Canine hip dysplasia development results in femoral neck modeling and an increase in thickness. The main objective of this work was to describe a femoral neck thickness index to quantify femoral neck width and to study its association with the degree of canine hip dysplasia using the *Fédération Cynologique Internationale* scoring scheme. A total of 53 dogs (106 hips) were randomly selected for this study. Two examiners performed femoral neck thickness index estimation to study intra- and inter-examiner reliability and agreement. Statistical analysis tests showed excellent agreement and reliability between the measurements of the two examiners and the examiners’ sessions. All joints were scored in five categories by an experienced examiner according to the *Fédération Cynologique Internationale* criteria, and the results from examiner 1 were compared between these categories. The comparison of mean femoral neck thickness index between hip dysplasia categories using the analysis of variance test showed significant differences between groups. These results show that femoral neck thickness index is a parameter capable of evaluating proximal femur bone modeling and that it has the potential to enrich conventional canine hip dysplasia scoring criteria if incorporated into a computer-aided diagnosis software.

**Abstract:**

The alteration in the shape of the femoral neck is an important radiographic sign for scoring canine hip dysplasia (CHD). Previous studies have reported that the femoral neck thickness (FNT) is greater in dogs with hip joint dysplasia, becoming progressively thicker with disease severity. The main objective of this work was to describe a femoral neck thickness index (FNTi) to quantify FNT and to study its association with the degree of CHD using the *Fédération Cynologique Internationale* (FCI) scheme. A total of 53 dogs (106 hips) were randomly selected for this study. Two examiners performed FNTi estimation to study intra- and inter-examiner reliability and agreement. The paired *t*-test, the Bland-Altman plots, and the intraclass correlation coefficient showed excellent agreement and reliability between the measurements of the two examiners and the examiners’ sessions. All joints were scored in five categories by an experienced examiner according to FCI criteria. The results from examiner 1 were compared between FCI categories. Hips that were assigned an FCI grade of A (n = 19), B (n = 23), C (n = 24), D (n = 24), and E (n = 16) had a mean ± standard deviation FNTi of 0.809 ± 0.024, 0.835 ± 0.044, 0.868 ± 0.022, 0.903 ± 0.033, and 0.923 ± 0.068, respectively (ANOVA, *p* < 0.05). Therefore, these results show that FNTi is a parameter capable of evaluating proximal femur bone modeling and that it has the potential to enrich conventional CHD scoring criteria if incorporated into a computer-aided diagnosis capable of detecting CHD.

## 1. Introduction

Canine hip dysplasia (CHD) is an inherited orthopedic disease predominant in large and giant dog breeds that causes lameness and disability. Phenotypic expression of CHD is influenced by genetic defects and environmental stresses that trigger hip joint laxity and incongruency, which often leads to bone modeling and progression to secondary osteoarthritis (OA) [1,2]. Molecular tests for the diagnosis of CHD have already been developed, but they still have not achieved acceptable diagnostic accuracy for the disease [3,4]. Radiography has remained the established imaging technology for diagnosing CHD, as it plays an important role in the selection of breeding stock with the aim of reducing the incidence of the disease in offspring [1,2]. In general, there is not a consistent relationship between clinical signs and radiographic joint changes [5,6]. The ventrodorsal hip extended (VDHE) view is recommended worldwide for CHD screening [7]. However, in young animals, a ventrodorsal hip stress view can also be used to evaluate the hip joint laxity [1,2]. Hip laxity is considered a main risk factor for CHD, but there are some important differences in the progression and final severity of CHD [5,6]. An image of good technical quality of the VDHE view requires radiographic images without pelvic tilting and with adequate femur extension and alignment [7]. However, in the last decades, despite the widespread use of CHD screening radiographs, the prevalence of the disease remains high in some breeds due to a number of factors. These include: variability between radiologists’ assessments, which is due to different levels of expertise among radiologists; screening systems that are not yet sufficiently standardized and strict, allowing for some subjectivity; the late appearance of unequivocal pathognomonic radiographic signs, at an age stage in which sometimes the dog has already entered the breeding pool; and the absence of evaluation of potential early hip joint changes in this view [8,9,10]. Therefore, the current incidence of CHD highlights the need to introduce new parameters to improve diagnosis, such as the hip congruency index, which has the potential to confer greater objectivity to the assessment of hip congruency, consequently benefiting overall diagnostic and scoring accuracy [11].

Worldwide, there are three main international entities for CHD scoring: the Fédération Cynologique Internationale (FCI), with implementation in the countries of continental Europe, the British Veterinary Association/The Kennel Club (BVA/KC), used mainly in the United Kingdom, and the Orthopedic Foundation for Animals (OFA), used in the United States of America [1,6,12]. All of these scoring systems place great emphasis on bone modeling and OA [12]. They also take a qualitative evaluation approach to these parameters, which leaves some margin for interpretation and error [8,13,14]. Early screening for CHD, commonly referred to as the PennHIP method, is based on distinct hip abnormalities. It assesses hip joint laxity by analyzing the femoral head separation from the acetabulum under stress by measuring the distraction index (separation distance divided by the radius of the femoral head) [1,2,6].

In CHD, early joint osteoarthritis and a consequent increase in joint fluid leads to incongruency, laxity of the soft tissue of the hip joint, and subluxation that results in abnormal stresses placed on the bony and soft tissue components of the joint. The joint capsule is attached to the margin of the acetabulum and around the femoral neck. The femoral neck is considered to be normal when its diameter narrows slightly directly below the head. The presence of biomechanical imbalance results in cartilage wear and tear and in the development of mechanosensitive pathways that drive proteases to initiate the mechanism of joint breakdown, subchondral and periosteal reaction, and new bone production in the capsule attachment area and neighboring tissues, particularly around the junction between the head and neck [15,16]. One of the main signs of CHD is the widening of the appearance of the femoral neck on a craniocaudal radiograph view due to osteophyte development in conjunction with the flattening of the femoral head, the former becoming progressively thicker until it is indistinguishable from the head in severe stages of CHD due to bony proliferation (exostoses) [17,18,19]. The FCI proposes a 5-grade classification system to represent the severity of the disease: A (normal), B (near normal/transition), C (mild), D (moderate), and E (severe) [6,12,16,17]. Current recommendations in CHD FCI scoring are to include Norberg angle measurement, joint space evaluation, congruence, osteoarthritic signs, and all aspects of hip joint changes, commonly referred as Brass’ method [17]. A previous study reports that the femoral neck thickness (FNT) is altered in hip joints classified as near normal (grade B) by the FCI system, adopting a slightly cylindrical shape. This morphological change becomes even more evident in hip joints classified as moderate grade (grade D) [17].

The main objective of this study was to create a new measurement method focused on alterations of the femoral neck as a means of determining proximal femoral changes associated with bone modeling and OA for CHD. For this purpose, we calculated the femoral neck thickness index (FNTi), an objective parameter that relates the minimal FNT to the diameter of the ipsilateral femoral head, and compared it to the different FCI grades. Our hypothesis was that there would be an association between FCI grades and FNTi, and that FNTi would increase with disease severity. To our knowledge, the FNTi’s association with the FCI grades has not been previously studied and could be integrated into a classification system as a parameter for evaluating bone modeling of the proximal femur.

## 2. Materials and Methods

This was a retrospective study based on the evaluation of VDHE views that were randomly selected from the Veterinary Teaching Hospital of the University of Trás-os-Montes and Alto Douro database and from the Danish Kennel Club database, obtained between 2010 and 2023. Recorded data included breed, sex, and weight. The inclusion criteria were dogs older than 12, 15, or 18 months (according to FCI recommendations for medium, large, and giant breeds) and VDHE views with adequate technical quality in terms of image (good bone contrast and spatial resolution) and pelvis positioning (femurs parallel to each other, patellae centred between the femoral condyles, and pelvic symmetry) for CHD scoring. VDHE views showing radiographic signs compatible with other hip or hindlimb diseases, such as bone fractures, previous surgeries, neoplasia, and knee osteoarthritis, were excluded. All five FCI categories were similarly represented in the sample. Due to the observational nature of this study, owner consent and ethical committee approval were waived.

The sample included 53 dogs of 13 different breeds: Portuguese Pointers (13/53, 25%), Estrela Mountain dogs (12/53, 23%), Transmontano Mastiffs (8/53, 15%), German Shepherd dogs (5/53, 9%), Labrador Retrievers (3/53, 9%), Bernese Mountain dogs (2/53, 4%), Barbado da Terceira (1/53, 2%), French Bulldog (1/53, 2%), German Shorthaired Pointer (1/53, 2%), Old Danish Pointer (1/53, 2%), Portuguese Sheepdog (1/53, 2%), Portuguese Water dog (1/53, 2%), and Rottweiler (1/53, 2%). There were 19 (36%) males and 34 (64%) females. The age ranged from 12 to 166 months, and the mean ± standard deviation (SD) was 31.87 ± 29.07 months. The average body weight was 36.1 ± 14.1 kg.

### 2.1. Radiographic Measurements

**Figure 1:**
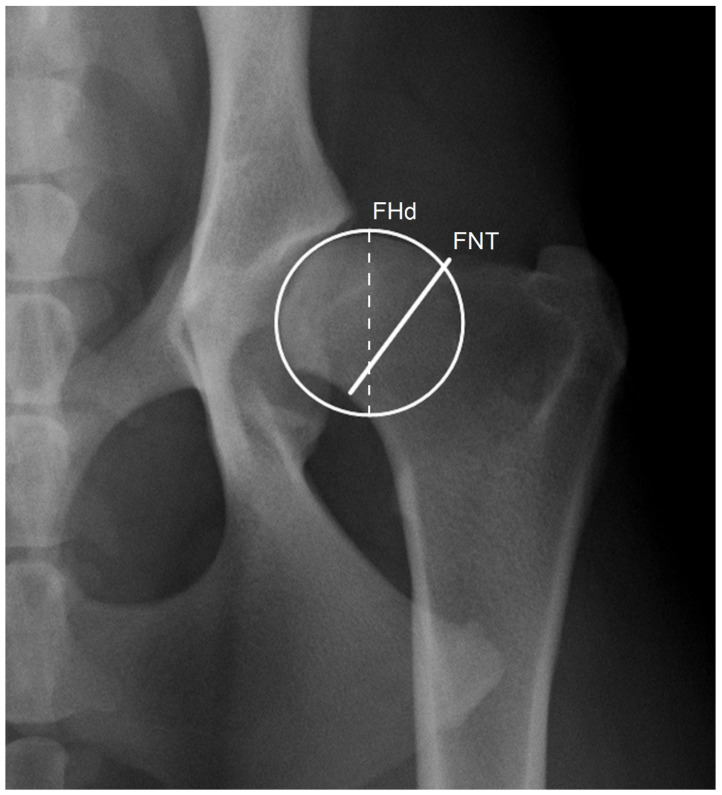
Detail of the left hip joint of a dog showing a circle delimiting the head of the femur with 31.5 mm diameter (FHd; dashed line) and a line joining the proximal and distal borders of the femoral neck to determine its thickness of 28.7 mm (FNT), corresponding to an index (FNTi) of 0.91.

The minimal FNT was measured by drawing a straight line, roughly perpendicular to the anatomical axis of the femoral neck, connecting the two closest points between the proximal and distal margins of the femoral neck in a VDHE view. The femoral head diameter was determined as a diameter of a circle outlining the margin of the femoral head (Figure 1). These measurements were performed by examiner 1 (E1) in two independent sessions (S1 and S2) to evaluate repeatability and by examiner 2 (E2) to test the reproducibility using specific DICOM viewer and editor software (Dys4Vet version 2.0, accessed between 1 October and 31 December 2022). The FNTi was determined by dividing the FNT by the femoral head diameter.

In order to associate FNTi with the five FCI grades for CHD, the hip joints were scored using FCI criteria: grade A (normal hip—Norberg angle (NA) > 105° and excellent congruency); grade B (borderline or transitional hip joint—NA around 105° and mild incongruency); grade C (slight CHD—NA around 100°, centre of femoral head outside of dorsal acetabular margin, and moderate incongruency); grade D (moderate CHD—NA > 90°, signs of osteoarthritis, and obvious incongruency); and grade E (severe CHD—NA < 90°, signs of osteoarthritis, and severe incongruency) [12,17]. The NA was measured between a line joining the centre of the circle encompassing the femoral heads and another line connecting each centre of the femoral head with the ipsilateral, effective cranial acetabular rim [20]. Some radiographic parameters related to the femoral head centre position and the dorsal acetabular margin and joint space were also considered for final FCI joint scoring [17].

The E1 and E2 measurements were performed by I.P. and P.F., respectively, and the hip FCI scoring was performed by M.G. in a single-blind fashion for each examiner (i.e., E1 and E2 were unaware of each FNTi measurement and FCI scores, and the FCI scorer was unaware of FNTi measurements).

### 2.2. Statistical Analysis

SPSS computer software (SPSS Statistics for Windows Version 27.0: IBM Corp., Armonk, NY, USA) was used to perform the statistical analysis.

Parametric tests were used for statistical analysis [21]. The paired *t*-test was used for comparison of duplicate E1S1–E1S2 measurements to evaluate repeatability, as well as E1S1–E2 measurements to evaluate reproducibility [22]. The Bland–Altman analysis and the intraclass correlation coefficient (ICC) were used to investigate intra- and inter-examiner agreement and reliability, respectively. In the Bland–Altman method, the 95% limits of agreement (LA) were calculated as the mean difference (*d*) ± 1.96 standard deviation (SD) [22,23,24,25]. Measurements were considered in agreement when the 95% confidence interval (CI) of the mean differences included zero and equivalent when the 95% upper and lower LA were small (irrelevant difference) [23,24]. The ICC was considered as poor, moderate, good/acceptable, and excellent reliability when the lower limit of 95% CI was <0.50, ≥0.50–0.75, ≥0.75–90, and ≥0.90, respectively [25,26]. Cohen’s d was used to measure the effect size when significant differences between measurements were registered: negligible < 0.20, small ≥ 0.20, medium ≥ 0.50, and large ≥ 0.80 [27].

The comparison of FNTi values of E1S1 measurement between FCI categories was performed using the Welch’s ANOVA, followed by the post hoc Games–Howell test. The null hypothesis was that there were no significant differences in the FNTi mean values between the FCI categories [28]. A *p*-value of <0.05 was considered statistically significant. The statistical analysis was performed considering each joint individually.

## 3. Results

**Figure 2:**
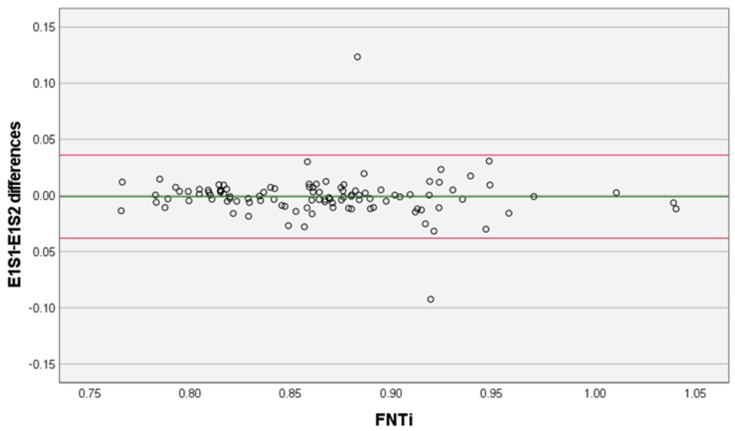
Examiner 1 differences between Sessions 1 and 2 (E1S1; E1S2). The green line represents the mean of the differences (−0.001) and the red lines represent the lower and upper 95% limits of agreement, −0.038 and 0.036, respectively. FNTi–femoral neck thickness index.

**Figure 3:**
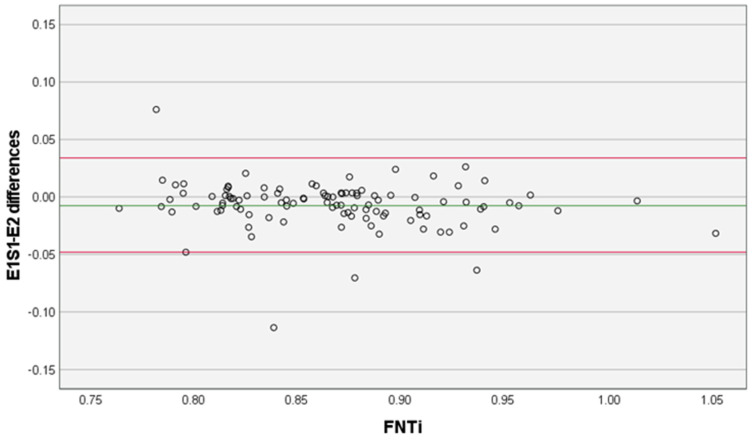
Examiner 1, Session 1 (E1S1) and Examiner 2 (E2) measurement differences. The green line represents the mean of the differences (−0.007) and the red lines represent the lower and upper 95% limits of agreement, −0.048 and 0.034, respectively. FNTi–Femoral neck thickness index.

Measurements were performed on 106 hip joints. The FNTi mean ± SD in E1S1 was 0.86 ± 0.06; in E1S2 it was 0.86 ± 0.06; and in E2 it was 0.87 ± 0.06. The main statistical analysis results related to the intra-examiner (repeatability) (*p* > 0.05 in paired *t*-test; ICC = 0.94 [95% CI, 0.92–0.96]; *d* ± SD −0.001 ± 0.019 [95% LA, −0.038, 0.036]) and inter-examiner (reproducibility) (*p* < 0.05 in paired *t*-test; ICC = 0.93 [95% CI, 0.90–0.95]; *d* ± SD −0.007 ± 0.021 [95% LA, −0.08,4 0.034]) measurements of FNTi are presented in detail in Table 1, Figure 2 and Figure 3.

**Figure 4:**
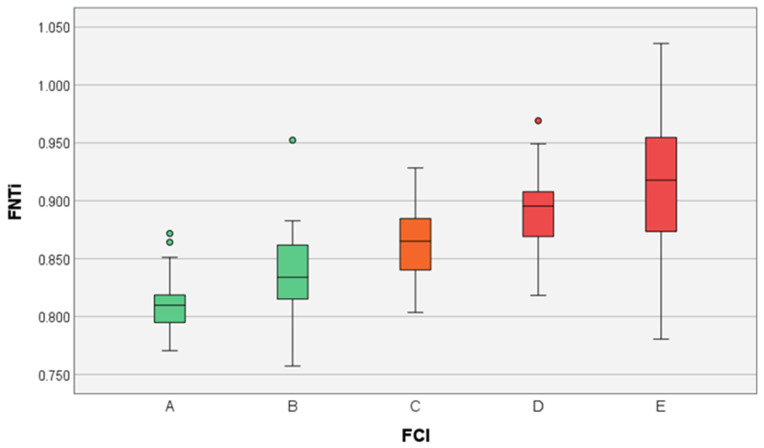
Box and whisker plot presenting the femoral neck thickness index (FNTi) classified in Fédération Cynologique Internationale (FCI) categories (A–E). The box plots show from bottom–up minimum data value, lower quartile value, median value, upper quartile value, maximum data value, and outliers (distant from the rest of the data).

A total of 19 (18%) hip joints were scored as FCI grade A, and the FNTi mean ± SD was 0.809 ± 0.024; 23 (21%) hip joints were scored as FCI grade B, and the FNTi mean ± SD was 0.835 ± 0.044; 24 (23%) hip joints were scored as FCI grade C, and the FNTi mean ± SD was 0.868 ± 0.022; 24 (23%) hip joints were scored as FCI grade D, and the FNTi mean ± SD was 0.903 ± 0.033; and 16 (15%) hip joints were scored as FCI grade E, and the FNTi mean ± SD was 0.923 ± 0.068. Data were assessed for normality using the Shapiro–Wilk test (p_A_ = 0.501; p_B_ = 0.275; p_C_ = 0.739; p_D_ = 0.983; p_E_ = 0.383), and Levene’s test indicated unequal variances among group samples (*p*_A,B,C,D,E_ < 0.05). Significant statistical mean differences using Welch’s ANOVA followed by the post hoc Games–Howell test were verified in FCI categories with means marked with different letter superscripts (*p* < 0.05) (Table 2, Figure 4).

## 4. Discussion

The objective of this study was to create a methodology that would allow a more objective radiographic evaluation of the changes that the proximal femur undergoes in CHD (namely, thickening of the neck), and to understand this relationship across the various FCI degrees of CHD in the VDHE view. The use of a parametric test in statistical analysis was based on the Central Limit Theorem, which states that in large sample sizes (n > 30), the distribution of standardized samples’ means tends to be normally distributed independently of the distribution of the population from where it originated [21]. The FCI scoring can be considered a dynamic system that has undergone regular updates over the years in order to harmonize CHD classifications in different countries and to improve the reliability of associating morphological alterations with the genetic profile of the animal. We highlight here the CHD panelist meetings of Dortmund 1991 and Copenhagen 2007 and 2022 [29].

In dysplastic hips, progressive bone modeling and osteophyte formation are induced by osteoarthritic pathways that result in subchondral and periosteal response with new bone production. Particularly around the junction between the head and neck, biomechanical stresses in the hip joint occur at a faster rate, and new bone and osteophytes are placed in some areas and reabsorbed in other areas of the femur and acetabulum [15]. These changes in the bone structure are concurrent with hip osteoarthritis [30]. Pinna et al. (2022) observed a significant increase in the thickness of the femoral neck in VDHE views of hips classified as grade B, a grade assigned to hips considered healthy and in which no osteoarthritic signs are visible [17]. In another study, Andronescu et al. (2015) studied the ratio between head volume and femoral neck volume on 3D computed tomography images of dogs at high risk of developing CHD (distraction index > 0.3) from 16 to 32 weeks of age and found a decrease in the ratio, even though the differences between values corresponding to OA severities were not statistically significant [18]. Therefore, the study of bone modeling and OA as two interrelated topics seems particularly important to us. We use the FNTi to get around the difficulty that exists in veterinary medicine to use absolute measures in anatomic measurements due to the different sizes of dogs. The relationship between measurements and the size of the femoral head is a strategy that has been successfully used previously for other purposes, such as the hip distraction index (mainly in young animals without severe bone changes) [1,6] and the hip congruency index [11]. In a previous study, the femoral neck width was related with the femoral neck length to create a widening index [31]. However, this methodology was not followed in this work because we think that the relationship between the length of the femoral neck and the size of the breed is much less studied and used than the diameter of the femoral head [6,11]. As such, there may be greater variability in the length of the femoral neck between breeds of similar sizes, which makes this parameter a less effective ratio measure. On the other hand, artificial intelligence is currently being introduced in digital image analysis [32,33], and since the identification of the femoral head seems to us to be an essential parameter in the application of artificial intelligence to the diagnosis of CHD, any index that resorts to its use can be more easily integrated in the near future for this purpose.

Bone modeling is admittedly one of the aspects of classification that is undervalued by the FCI criteria. In some iterations of the FCI criteria [34], morphological changes of the proximal femur are only explicitly mentioned in the most severe degree of dysplasia (grade D), pointing out the characteristic mushroom-like appearance that the femoral head assumes. Other changes related to bone modeling of the proximal femur have been overlooked and are presumably relegated to the topic of “osteoarthritic signs”, addressed with a yes/no question [34,35], which makes the evaluation less accurate. The BVA/KC uses a phenotypic evaluation criteria based on a points system, addressing changes in bone shape from minor modeling up to severe OA [19]. However, it is still a qualitative scoring system, so there is also some subjectivity in the analysis because it ends up being dependent on the level of experience of the examiner. Taking this into consideration, there is a perceived need for novel approaches that grant more objectivity to the assessment of bone modeling and, in addition, CHD scoring.

Our results showed that the methodology behind the FNTi had excellent reliability and agreement. Given the results of the paired *t*-tests and Bland-Altman plots with the mean differences near zero and the narrow 95% CI, there was no evidence of bias between the examiners’ measurements, and they can be considered statistically similar. The intra-examiner ICC was 0.94, and the lower bound of the 95% CI was 0.92, which translates to excellent reliability. This indicates adequate repeatability and reproducibility of the described FNTi determination methodology. Significant mean differences between FNTi examiners’ measurements were observed in the paired samples *t*-test. However, the recorded effect size was negligible (*d* = 0.14) and, considering the corresponding Bland–Altman plot (Figure 3), the 95% LA can be interpreted as clinically small because 95% of all calculated inter-examiner differences lie in the short range of −0.048 to 0.034. Furthermore, the inter-examiner ICC shows excellent reliability. This shows that even though the two examiners themselves produce somewhat different values, their measurements are clearly related and functionally consistent. Both examiners had a similar amount of practice time with the method beforehand, so no conclusions can be drawn based on experience. On the other hand, one would expect the mean differences to be greater among bigger FNTi mean values due to the subjectiveness of the annotation imposed by exostosis and/or osteophyte formation in the concave fossa, making the bone contours more intricate, which could have possibly caused disparities between sessions and examiners. Ultimately, by analyzing the two diagrams (Figure 2 and Figure 3), we can see that the mean difference values do not increase or decrease in proportion to the mean FNTi values, thus concluding that there is no proportional bias.

The Welch’s ANOVA of FNTi values in FCI categories revealed a statistically significant main effect (*p* < 0.001), indicating that not all FCI categories had the same mean FNTi value. Post hoc comparisons using the Games–Howell post hoc procedure were conducted to determine which pairs of the five categories’ means differed significantly. The results indicate that different FCI categories have mean FNTi values that increase gradually with the degree of severity of the disease: grade A hips had a statistically significant lower mean FNTi value than grades C, D, and E (*p* < 0.05); grade B had significantly lower mean FNTi value than grades D and E (*p* < 0.05); and grade C had significantly lower mean FNTi value than grade E (*p* < 0.05). Therefore, the null hypothesis is rejected, which supports our assumption that FNTi changes with FCI CHD grades. This parameter can help distinguish between CHD grades, potentially improving FCI scoring criteria. However, there is some overlap in the FNTi values corresponding to the different FCI categories, specifically between adjacent categories. This is one of the reasons that an assessment solely dependent on this is ambiguous and, therefore, impractical. The “whiskers” of the box plot corresponding to category E stretch over a wider range of values than the other box plots, overlapping almost every other category. This can be explained by how long hip subluxation has been in place and by the difficulty of adequate delimitation of the femoral head for its diameter measurement in joints with very severe bone osteoarthritic deformations. In severe cases with long-term subluxation, the surface of the femoral head cartilage at the non-articulated margin of the head and neck becomes thickened due to a lack of contact with the opposite acetabular surface [15]. Hence, a long-term subluxated hip favors the appearance of the so-called mushroom head deformity, which inflates the diameter of the femoral head compared to the neck thickness, thus producing lower FNTi values pertaining to category E, values that lie in the lower “whisker” of the box plot. We strongly advocate that this parameter should never be used by itself to classify hips, but rather in complementarity with other parameters. On the other hand, given the recognized variability that exists in the progression of CHD, femoral neck bone modeling, and femoral head size, it is expected that more robust FNTi results will be obtained if a study is performed using only one breed in the sample [5].

Since our study sample was sourced from two different databases and included a variety of breeds, the results can be more easily extrapolated to general canine populations at risk of CHD. Nonetheless, as a limitation of this study, it is important to note that some breeds prone to CHD are clearly over-represented (Portuguese breeds); others, on the contrary, are under-represented (Labrador Retriever and Rottweiler). As such, in the future, there is a need to conduct more studies on a wider range of breeds and patients.

## 5. Conclusions

This study describes a methodology that allows for the evaluation of bone modeling of the proximal femur in the VDHE view, which can be used in the future with confidence as a criterion for CHD scoring. The FNTi shows adequate intra- and inter-examiner measurement agreement and reliability. Mean FNTi values are gradually higher in the different FCI categories, with statistically significant differences. The FNTi method shows potential to make CHD classification more objective if incorporated as a scoring criterion, and it is also promising in view of its application through a computer-aided diagnosis capable of autonomously identifying and classifying CHD.

## Figures and Tables

**Table 1:** Statistical analysis of the intra- and inter-examiner agreement and reliability of the FNTi in measuring 106 hips.

Comparison		Paired *t*-Test *p*-Value	Cohen’s *d*	ICC [95% CI]	*d* ± SD	95% LA	
						Lower Bound	Upper Bound
Intra- examiner	E1S1–E1S2	0.583	--	0.94 [0.92,0.96]	−0.001 ± 0.019	−0.038	0.036
Inter- examiner	E1S1–E2	<0.001	0.14	0.93 [0.90,0.95]	−0.007 ± 0.021	−0.048	0.034

Abbreviations: CI, confidence interval; *d*, mean difference; E, examiner; FNTi, femoral neck thickness index; ICC, intraclass correlation coefficient; LA, limits of agreement; S, session; SD, standard deviation.

**Table 2:** Statistical descriptive analysis of the femoral neck thickness index by FCI categories.

FCI Categories	*N*	Mean *	SD	Mean 95% CI		Min	Max
				Lower Bound	Upper Bound		
A	19	0.809 ^a^	0.024	0.797	0.823	0.771	0.864
B	23	0.835 ^a,b^	0.044	0.812	0.859	0.757	0.952
C	24	0.868 ^b,c^	0.022	0.856	0.880	0.831	0.918
D	24	0.903 ^c,d^	0.033	0.886	0.921	0.841	0.969
E	16	0.923 ^d^	0.068	0.887	0.959	0.781	1.036

* Means with different superscripts are statistically different (*p* < 0.05) in the post hoc Games–Howell test that followed Welch’s ANOVA. Abbreviations: CI, confidence interval; FCI, Fédération Cynologique Internationale; SD, standard deviation.

## Data Availability

Not applicable.
